# Trained immunity in cancer immunotherapy and vaccine development: mechanisms, evidence gaps, and translational opportunities

**DOI:** 10.3389/fimmu.2026.1939256

**Published:** 2026-09-28

**Authors:** Lian Yang, Jianfeng Liu, Shufei Zhang, Yong He, Jiaxu Chen, Donghui Tan

**Affiliations:** 1 Affiliated Hospital of Xiangnan University, Chenzhou, Hunan, China; 2 Guangzhou Key Laboratory of Formula-Pattern of Traditional Chinese Medicine, School of Traditional Chinese Medicine, Jinan University, Guangzhou, China; 3 Department of Gynecology and Obstetrics, Renmin Hospital of Wuhan University, Wuhan, China; 4 School of Traditional Chinese Medicine, Beijing University of Chinese Medicine, Beijing, China

**Keywords:** cancer immunotherapy, cancer vaccine, innate immunity, metabolic reprogramming, trained immunity

## Abstract

Breakthroughs in cancer immunotherapy have been overwhelmingly driven by advances in adaptive immunity, leaving the therapeutic potential of innate immunity largely untapped, a gap in the current treatment paradigm. Trained immunity, defined by the durable, mitotically maintained metabolic and epigenetic memory that innate immune cells acquire after transient stimulation, offers a new approach to addressing this deficit. Here we survey the triggering signals, cellular heterogeneity, and molecular circuitry that govern trained immunity within the tumor microenvironment. We dissect its dualistic character: the mechanistic basis by which trained immunity can either restrain or promote tumor progression, and what this ambivalence means for the design of next-generation immunotherapies. These insights inform a growing pipeline of trained-immunity-based cancer vaccines, which we categorize into four engineered platforms, recombinant BCG, β-glucan nanoformulations, lipid nanoparticle (LNP)-mRNA bifunctional vaccines, and commensal-derived adjuvants. We further propose that vaccine efficacy may depend in part on host immune context, and outline stratification strategies and dosing schedules that may need to be adjusted according to individual immune status, pending clinical validation. In aggregate, evidence from human bladder cancer, preclinical tumor models, and non-tumor trained immunity studies suggests that trained immunity could elevate innate immunity from a supporting role toward an independent therapeutic axis, although most of its direct cancer applications remain at the preclinical or conceptual stage.

## 1 Introduction

Immune checkpoint inhibitors (ICIs) have transformed outcomes for selected patients and tumor types, yet clinical benefit remains heterogeneous across solid tumors and primary or acquired resistance is common (1, 2). Therapeutic strategies centered on adaptive immunity, especially T cells, have achieved meaningful responses but face persistent ceilings in many tumor settings; these limitations do not reflect a fundamental opposition between adaptive and innate immunity—both compartments are typically interdependent—but rather the fact that the innate immune compartment in many tumors remains largely silent. Within the tumor microenvironment (TME), M2-polarized tumor-associated macrophages, expanded myeloid-derived suppressor cells, and functionally impaired dendritic cells collectively form a potent immunosuppressive network that T-cell-directed interventions alone struggle to overcome (3, 4).

Trained immunity offers a conceptual way around this impasse. In 2011, Netea and colleagues demonstrated that innate immune cells, after a brief stimulus, can develop a durable, enhanced functional state through metabolic reprogramming and epigenetic remodeling (5, 6). Unlike approaches that act predominantly on mature effector cells, trained immunity can operate at both central and peripheral levels. Central training may involve bone marrow hematopoietic progenitors and altered myeloid output, whereas peripheral training can persist in circulating monocytes and tissue-resident macrophages through durable metabolic and epigenetic reprogramming (7, 8). Four decades of clinical experience with intravesical Bacillus Calmette-Guérin (BCG) for bladder cancer provide a proof-of-concept that innate immune modulation can yield durable clinical benefit in human oncology (3, 6). Trained immunity is a plausible and increasingly supported contributor to BCG efficacy, rather than the sole established mechanism. BCG treatment of non-muscle-invasive bladder cancer (NMIBC) involves multiple immunological processes, including local inflammation, urothelial and APC activation, granulocyte and macrophage recruitment, NK cell activity, T-cell and antigen-specific responses, and trained immunity, alongside strain-, dose-, exposure-duration, and host-genetic variables (3, 6, 9). In summary, the clinical efficacy of BCG in bladder cancer arises from the convergence of local inflammation, specific T-cell-mediated immunity, and trained immunity, rather than from trained immunity alone.

The mechanistic sections that follow are included to provide the biological rationale for the translational anticancer interventions discussed later; throughout, we distinguish direct tumor evidence from evidence extrapolated from non-tumor trained-immunity models and from author-proposed, testable hypotheses. With this backdrop, we survey the landscape of trained immunity in tumor immunology around three central questions (1): How do innate immune cells rewire their functional programs through coupled metabolic and epigenetic remodeling? (2) How do tumors co-opt trained immunity to fuel their own progression? (5) Can trained immunity be engineered to amplify antitumor responses? Our goal is to link the mechanisms of trained immunity to testable intervention strategies and identify steps toward clinical translation.

## 2 Scope and approach

This narrative review was conducted using a structured, though not systematic, literature search. We searched PubMed/MEDLINE, Embase, Web of Science, and Google Scholar for publications from 2011 to June 30, 2026 (the final search date), using combinations of the following keywords: ‘trained immunity’, ‘innate immune memory’, ‘trained immunity cancer’, ‘trained immunity tumor microenvironment’, ‘trained immunity vaccine’, ‘β-glucan’, ‘BCG’, ‘STING’, ‘LNP’, ‘mRNA vaccine’, ‘myeloid reprogramming’, and ‘epigenetic remodeling’. The search was restricted to English-language publications. Inclusion criteria were: (i) peer-reviewed original research articles or comprehensive reviews directly addressing trained immunity mechanisms, trained immunity in cancer, or trained immunity-based interventions; (ii) studies published after 2011 (the year trained immunity was formally described), with landmark earlier studies included for historical context. Exclusion criteria were: (i) preprint manuscripts not subsequently peer-reviewed; (ii) studies on conventional adaptive immune memory without innate immune relevance; (iii) duplicate reports of the same dataset. Primary research studies were distinguished from reviews by examining the article type designation and methodology section; when both original data and review elements were present, the article was classified by its primary contribution. When contradictory evidence was identified for the same conclusion, both supporting and contradicting studies were noted in the text, and the discrepancy was discussed rather than selectively reporting one side. We applied the operational definition of trained immunity as described in the Introduction and used the five-level evidence hierarchy described in the ‘Evidence hierarchy framework’ section to classify claims throughout the review. No formal PRISMA flow diagram or systematic meta-analysis was performed; evidence was synthesized narratively, with an emphasis on identifying conceptual frameworks, preclinical proof-of-concept, and available clinical data. As a narrative rather than systematic review, this approach carries inherent methodological limitations. The literature search, while structured, was not exhaustive; study selection was based on author judgment rather than predefined inclusion and exclusion criteria, introducing potential selection bias. The absence of dual independent screening, formal quality assessment of individual studies, and quantitative evidence synthesis represents a methodological constraint. Consequently, the synthesis presented herein reflects a conceptual overview and expert perspective rather than a comprehensive or unbiased enumeration of all available evidence.

## 3 Trained immunity in the tumor context

A key feature of trained immunity is that innate immune cells, following a transient stimulus, undergo persistent metabolic and epigenetic reprogramming; even after the stimulus subsides, these cells retain a heightened response capacity, the functional equivalent of immunological memory within the innate system (6). This process must be distinguished from three related but mechanistically distinct phenomena. Priming transiently upregulates gene transcription without establishing long-term memory. Tolerance suppresses secondary responses, the polar opposite of the response-enhancing character of trained immunity. Differentiation requires sustained microenvironmental signals and cannot be initiated by brief stimulation alone. The hallmark of trained immunity is that global transcription may largely return toward baseline after stimulus withdrawal, yet epigenetic marks persist, enabling an accelerated, amplified response upon subsequent challenge (10).

Trained-immunity-mediated long-term reprogramming operates at two tiers, central and peripheral, that together shape the immunological trajectory of the TME. Central training occurs in bone marrow hematopoietic stem and progenitor cells (HSPCs), where circulating cytokines and metabolic cues bias hematopoiesis toward myeloid differentiation, sustaining systemic immune effects that persist for mont (4, 11, 12, 13). Peripheral training, by contrast, acts on circulating monocytes and tissue-resident macrophages through cell-autonomous regulatory mechanisms. Unlike the better-characterized infection-driven trained immunity, the tumor microenvironment—extrapolated from non-tumor trained immunity models—continually exposes innate cells to a mixture of damage-associated molecular patterns (DAMPs), metabolites, and microbial products; the same reprogramming pathways can, depending on local context, mediate either antitumor or pro-tumor outcomes, a bidirectional phenotype that remains largely to be validated in human tumors.

Before considering tumor-specific applications, it is important to distinguish canonical trained immunity from acute innate priming, tolerance, and differentiation. These related phenomena can share cytokine or transcriptional features, but differ in stimulus history, persistence, recall behavior, and the requirement for durable metabolic or epigenetic reprogramming. To maintain conceptual precision throughout this review, **Table 1** summarizes the essential criteria that distinguish bona fide trained immunity from related but distinct phenomena. Studies not yet meeting all criteria are described using the terms “innate priming,” “memory-like response,” or “trained-immunity-like effect,” as appropriate (**Figure 1**).

**Table 1:** Essential criteria for defining trained immunity.

Criterion	Description	Distinguishes from
1. Transient initial stimulus	The inducing stimulus is brief or episodic, not continuous	Persistent activation, chronic stimulation
2. Persistence after stimulus withdrawal	Functional changes persist after the stimulus is removed	Acute innate priming, transient inflammation
3. Augmented response upon restimulation	An enhanced or altered response is observed upon secondary challenge	Baseline tolerance, unresponsiveness
4. Metabolic or epigenetic reprogramming	Demonstrable changes in metabolic pathways and/or chromatin modifications (e.g., H3K4me3)	Cytokine-driven activation without memory
5. Progenitor reprogramming (for short-lived cells)	For short-lived effector cells (e.g., neutrophils), reprogramming of HSPCs or progenitors should be demonstrated	Mature cell-only functional changes

**Figure 1:**
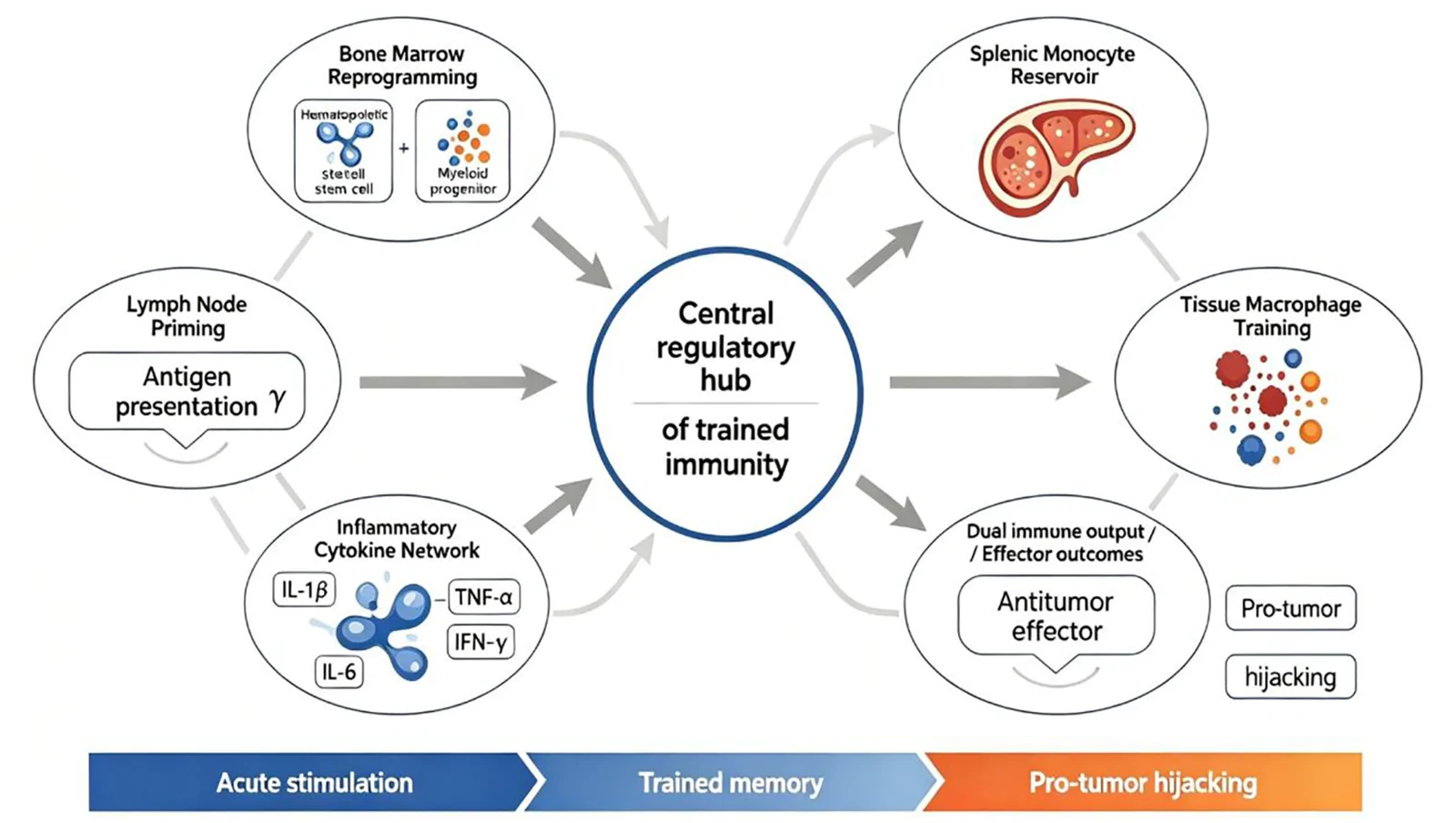
Central regulatory hub of trained immunity. The trained immune response integrates inputs from bone marrow reprogramming, splenic monocyte reservoirs, lymph node priming, inflammatory cytokine networks, and tissue macrophage training, ultimately producing dual immune outputs that can be directed toward antitumor effector function or pro-tumor hijacking.

## 4 Endogenous triggers of trained immunity in the tumor context

Diverse signals drive tumor-associated trained immunity, and understanding these triggers provides a rationale for vaccine adjuvant design and dosing-timing decisions.

### 4.1 Tumor microenvironment-derived endogenous damage-associated molecular patterns

Oxidized low-density lipoprotein (oxLDL) is a well-characterized endogenous inducer of trained immunity in non-tumor *in vitro* and atherosclerosis models. Brief oxLDL exposure can produce persistent pro-inflammatory functional reprogramming in monocyte-derived macrophages, including enhanced cytokine production and foam-cell-associated responses (13, 14). In these systems, signaling through pattern-recognition and lipid-uptake pathways, including TLR2, TLR4, and CD36, contributes to metabolic reprogramming. Whether comparable oxLDL-driven training occurs within the human TME remains unknown. The oxidized lipid landscape of tumors is heterogeneous and coexists with immunosuppressive mediators such as IL-10, TGF-β, PGE_2_, and adenosine; how these signals jointly influence myeloid training or polarization remains to be established (15). Accordingly, oxLDL should be viewed as a biologically plausible candidate training signal in tumors, not as a demonstrated driver of trained immunity in human cancer.

Beyond oxidized lipids, DAMPs released from dying tumor cells activate innate-sensing pathways within the TME. Their potential to establish canonical trained immunity is inferred largely from non-tumor inflammation and infection models; tumor-specific evidence for durable, restimulable training remains limited. HMGB1 signals through TLR2, TLR4, and RAGE; extracellular ATP acts via P2X7; HSP70 and HSP90 engage TLR4, all activating downstream axes that overlap substantially with canonical trained immunity pathways (16). In addition, hypoxia and lactic acidosis within the TME supply alternative, PRR-independent routes to training induction: hypoxia stabilizes HIF-1α, remodeling the glycolytic phenotype of myeloid cells (17), while lactate directly remodels chromatin state through histone lactylation (18). These non-canonical mechanisms, while mechanistically plausible, have been characterized exclusively in non-tumor and preclinical models; their operation within the solid tumor microenvironment remains hypothetical. Direct evidence linking hypoxia- or lactate-driven histone lactylation to trained immunity in human tumors is currently lacking, and these pathways should be regarded as candidate mechanisms requiring validation in tumor-specific contexts. Moreover, the window through which trained immunity could be hijacked by the TME, if these pathways are confirmed, would be substantially wider than currently demonstrated (19).

### 4.2 Therapy-induced trained immunity signals

Antitumor therapies, while killing tumor cells, release damage signals that can serve as spontaneous triggers of innate priming; whether they induce genuine trained immunity depends on demonstration of durable metabolic-epigenetic memory and, for short-lived cells, progenitor reprogramming. Chemotherapy agents such as anthracyclines and oxaliplatin induce immunogenic cell death (ICD), releasing calreticulin, HMGB1, and ATP; the downstream TLR4-HMGB1 and P2X7-ATP signaling axes overlap substantially with canonical trained immunity pathways (20). It remains a testable hypothesis that peripheral ICD-derived signals could contribute indirectly to central HSPC training after chemotherapy. Current literature supports ICD-associated innate activation, but does not establish a causal relay from tumor-derived DAMPs to bone-marrow progenitor reprogramming or to durable antitumor immune memory in patients (21). Radiotherapy can induce local ICD and systemic immune effects, including abscopal responses in selected settings; however, a specific causal role for trained-immunity reprogramming or systemic myeloid-reservoir remodeling has not been established and should be treated as a hypothesis rather than an inferred mechanism (22). Immune checkpoint inhibitors (ICIs) exhibit a more complex, bidirectional relationship with trained immunity: ICIs, by relieving T-cell inhibition, amplify the antitumor function of pre-activated T cells and can synergize with trained immunity (23); yet non-response to ICIs in a subset of patients may stem from myeloid cells that have already been stably reprogrammed into a pro-tumor phenotype under chronic tumor signaling, in which case restoring T-cell function alone cannot reverse the suppressive architecture at the innate immune level (24, 25).

These observations suggest a hypothesis: antitumor therapies may generate unexpectedly durable immune effects through the induction of trained immunity, although many of these signals remain at the level of innate priming or persistent activation until HSPC reprogramming is demonstrated. The upstream cascade of chemotherapy-induced ICD releasing DAMPs that engage PRRs has been extensively validated (20, 21), but gaps remain in the relay from peripheral DAMP release to bone marrow HSPC reprogramming. Whether DAMPs can effectively infiltrate the bone marrow microenvironment, what concentrations are functionally relevant *in situ*, and which intermediary mediators bridge the distance between peripheral release and central training all lack systematic experimental evidence. Longitudinal clinical cohort studies that integrate trained-immunity-relevant biomarkers represent the most direct route to testing this still-hypothetical relay.

## 5 Cellular diversity in trained immunity

### 5.1 Monocytes and macrophages: the canonical training carriers

The monocyte-macrophage lineage is the most thoroughly characterized cellular compartment in trained immunity research. Single-cell transcriptomic profiling stratifies human peripheral blood monocytes into three subsets (non-responder, chemokine-enhanced, and hyper-responsive), and this stratification persists for at least three months after BCG vaccination (26). In preclinical tumor models, the training status of tumor-associated macrophages (TAMs) sculpts the local immune landscape: β-glucan-induced training can lock TAMs into a stable M1-like antitumor phenotype (M1/M2 is a simplified binary designation; macrophage polarization *in vivo* is continuous and context-dependent) (23), whereas TME-derived signals drive TAMs toward immunosuppression and pro-angiogenic polarization through an IL-6-STAT3 autocrine loop (27). Identical myeloid precursors respond oppositely to training signals in peripheral blood and the TME. Clinically, measuring trained immunity only from circulating monocytes cannot capture macrophage reprogramming localized within tumor tissue (28, 29).

Inter-individual variability in training responsiveness raises a further concern: if a subset of patients is hypo-responsive or non-responsive at baseline, intervention strategies built around boosting trained immunity may be targeting the wrong population from the outset. Yet trainability is not a fixed trait. Tumor burden, host metabolic state, gut microbiota composition, and prior treatment history can all dynamically shift the response threshold of myeloid cells. This means that a low-trainability state is potentially reversible. Evidence from *in vitro* models indicates that AMPK agonists and DNMT or HDAC inhibitors can remodel the chromatin landscape of myeloid cells; if this concept can be validated *in vivo* and in human tumor settings, enhancing immune trainability would itself become a new intervention dimension, independent of any specific training inducer (30). While monocytes and macrophages serve as the canonical training effectors, their short circulating lifespan and continuous replenishment from bone marrow progenitors point to upstream cellular reservoirs, beginning with the most abundant circulating innate cell, the neutrophil.

### 5.2 Neutrophils: a new frontier in trained immunity

Neutrophils, owing to their short circulating lifespan, were long considered incapable of establishing long-lasting innate immune memory. This view has been overturned by several recent studies, yet neutrophil functional changes in the tumor context must be distinguished across four layers: (i) short-term functional alterations in mature circulating neutrophils; (ii) bone marrow granulopoiesis reprogramming; (iii) HSPC-mediated long-term changes; and (iv) neutrophil extracellular trap (NET) formation per se. Following BCG vaccination in healthy humans, circulating neutrophils develop a trained-immunity-like phenotype (meeting criteria 2 and partially 4, but not yet 5) that persists for months, marked by sustained upregulation of activation markers such as CD11b and genome-wide enrichment of H3K4me3 modifications (31); because neutrophils are short-lived, this persistence may reflect continuous replenishment from reprogrammed progenitors (bone marrow progenitor rewiring) rather than neutrophil-autonomous memory. In animal tumor models, the N1/N2 paradigm (similar to M1/M2 macrophage binary classification, N1/N2 is also an oversimplified grouping for context-dependent neutrophil functions) is linked to the direction of innate priming. By contrast, under chronic tumor stimulation, persistent NET release facilitates metastatic seeding, a pattern more consistent with persistent activation than canonical trained immunity (32). Neutrophil-targeted strategies must therefore balance antitumor effector priming against pro-metastatic NET release and N2-like polarization. The rapid turnover of neutrophils and their NET-dependent biology point to a further layer of innate memory: the longer-lived NK cell compartment, which couples direct cytotoxicity with immunological memory.

### 5.3 NK cells: innate immune memory pioneers

NK-cell memory-like responses are discussed here under the broader umbrella of innate immune memory, but their molecular basis may differ from classical monocyte/macrophage trained immunity. These phenomena are classified as “memory-like responses” rather than bona fide trained immunity, as they do not fully satisfy all five criteria in **Table 1** (notably, criterion 4 regarding metabolic reprogramming is incompletely characterized). Multiple NK memory-like states have been described, including cytokine-induced memory-like NK cells, CMV-associated adaptive NK cells, antigen-specific NK memory, and BCG-associated functional enhancement; these phenomena share mechanistic overlap with myeloid trained immunity but are not fully equivalent. NK cells serve as a critical link between innate and adaptive immune responses. BCG vaccination in healthy humans induces long-lasting adaptive-like NK memory, marked by enhanced IFN-γ secretion and degranulation-mediated cytotoxicity (33). Mouse models have further shown that NK cells with memory-like properties exhibit stronger cytotoxicity against tumor cell lines, an effect closely tied to the functional specialization of NK cell subsets (34). In the TME, however, prolonged exposure to high concentrations of IL-15 and TGF-β continuously remodels the activation state of NK cells, driving progressive exhaustion that ultimately erodes their antitumor killing capacity. While NK cells connect innate cytotoxicity and adaptive-like memory, the initiation of adaptive antitumor immunity critically depends on antigen-presenting cells, among which dendritic cells acquire qualitatively distinct functional properties following training.

### 5.4 Dendritic cells: training-enhanced antigen presentation

Following training stimuli, DCs coordinately upregulate surface MHC-I, MHC-II molecules, and CD80/CD86 costimulatory molecules, enabling efficient priming of tumor antigen-specific T cell responses (35). Trained and activated DCs secrete abundant IL-12 and IL-18, steering naive CD4+ T cells toward Th1 functional polarization while simultaneously amplifying CD8+ T cell cytotoxic effector function, a regulatory mechanism that provides theoretical support for novel cancer vaccine design.

A common limitation across the cellular compartments discussed above (monocytes, macrophages, neutrophils, NK cells, and dendritic cells) is the disproportionate reliance on *in vitro* models and non-tumor *in vivo* systems: direct evidence from human tumor tissues remains scarce for each of these lineages, and most mechanistic conclusions await validation in prospective oncology cohorts. Closing the gap between *in vitro* training paradigms and the complexity of the human TME is a prerequisite for translating cellular insights into clinical biomarkers or therapeutic strategies. The functional states of these mature effector cells are ultimately sustained by a deeper cellular reservoir, highlighting the central importance of HSPCs.

### 5.5 Hematopoietic stem and progenitor cells: the long-term reservoir of trained immunity

HSPCs constitute the central reservoir that sustains systemic, long-lasting trained immunity (36). BCG-induced epigenetic marks in these progenitors are stably propagated to progeny across multiple rounds of self-renewal through a C/EBPβ-dependent mechanism (37), endowing differentiated myeloid cells with enhanced immune function that persists for months. In cancer therapy, the significance of central HSPC training is conceptual: in principle, durable bone marrow progenitor reprogramming could sustain long-term myeloid immune surveillance, but whether a single intervention provides clinically meaningful protection during postoperative recurrence remains unknown. Stromal cell trained immunity and intergenerational innate memory have only been validated in non-oncologic models and bear minimal relevance to tumor vaccine development (38, 39). The output of trained HSPCs encompasses not only protective myeloid effectors but also, when training signals become chronic or dysregulated, immunosuppressive cell types including myeloid-derived suppressor cells (MDSCs).

### 5.6 Trained myelopoiesis, MDSCs and emergency myelopoiesis

Building on the HSPC-centered framework established above, we now address a critical boundary question: whether trained myelopoiesis can cross into pathological emergency myelopoiesis, a distinction with direct implications for the temporal dosing of central training strategies. Myeloid-derived suppressor cells (MDSCs) have been repeatedly mentioned in the preceding sections, yet they deserve a focused discussion because their biology sits at the intersection of trained myelopoiesis and pathological emergency myelopoiesis. Tumor-derived G-CSF, GM-CSF and IL-6 skew HSPC differentiation toward MDSC lineages in preclinical models (40, 41), biasing granulocytic and monocytic progenitors toward immunosuppressive fates (G-MDSCs and M-MDSCs) (41, 42). This recapitulates the same IL-6/STAT3 axis driving TAM immunosuppressive polarization (Section 5.1). Such expansion is functionally linked to impaired T-cell responses and accelerated tumor progression in animal models.

The conceptual boundary between trained immunity and pathological emergency myelopoiesis is especially important here. Emergency myelopoiesis is a physiological response to acute stress that rapidly expands myeloid output; when it becomes chronic and tumor-driven, it can evolve into a pathological state characterized by sustained MDSC expansion and immune suppression (25, 43). Whether this chronic state should be labeled trained immunity remains debated. Strictly speaking, canonical trained immunity requires a transient stimulus followed by a durable, altered response with persistent metabolic and epigenetic remodeling. In contrast, tumor-driven MDSC expansion often depends on continuous cytokine stimulation and lacks a clear quiescent phase, making it more accurately described as persistent activation or pathological emergency myelopoiesis rather than bona fide trained immunity.

This distinction has direct therapeutic implications for central training strategies. Therapeutic central training, aimed at generating long-lived antitumor myeloid cells, must be carefully dosed and timed to avoid pushing HSPCs toward MDSC differentiation. Preclinical evidence suggests that the same trained-immunity-inducing stimuli can produce either antitumor or pro-tumor outcomes depending on stimulus duration, intensity, and host context (Section 7.2) (25, 43). To minimize the risk of MDSC expansion, central training should be episodic rather than continuous, preferentially activate antitumor transcriptional programs (for example, IRF5/8-polarized myelopoiesis), and be combined with agents that block MDSC recruitment or suppressive function, such as anti-IL-6 or anti-G-CSF interventions (40, 44). Monitoring peripheral MDSC dynamics during therapy may serve as an early pharmacodynamic indicator of whether central training is shifting toward an immunosuppressive trajectory. The temporal window of HSPC training determines whether the output is protective or immunosuppressive, making MDSC monitoring a predictive pharmacodynamic biomarker for any central-training-based vaccine regimen. This temporal dosing logic echoes the intermittent BCG and β-glucan administration schedules discussed in Section 8, where episodic rather than continuous stimulation avoids pushing trained myelopoiesis into pathological emergency hematopoiesis.

The cellular diversity landscape outlined above, which includes monocytes, macrophages, neutrophils, NK cells, dendritic cells, HSPCs, and MDSCs, reveals a design principle: distinct vaccine platforms should be precisely matched to their effector cell tiers. HSPC-targeting vectors correspond to central training, whereas myeloid-cell-focused delivery engages peripheral effectors (45).

The next section dissects the core molecular regulatory networks of trained immunity, providing actionable targets for vaccine amplifier design.

## 6 Intracellular molecular mechanisms of trained immunity

This section focuses on the core molecular mechanisms of tumor-associated trained immunity, organized across four levels: metabolic-epigenetic coupling, central HSPC training, TME metabolic signal regulation, and local TAM training. It concludes with an integrative model that distills translational implications.

### 6.1 Metabolic-epigenetic coupling: the core driver of trained memory

The molecular logic of trained immunity is metabolic reprogramming driving epigenetic memory (28). Functional blockade of the TCA cycle at succinate dehydrogenase (SDH) and fumarate hydratase (FH) leads to fumarate accumulation, which inhibits KDM5 family demethylases and thereby preserves the H3K4me3 activation mark at promoters of pro-inflammatory genes such as TNF and IL6 (46, 47). Succinate, in parallel, inhibits prolyl hydroxylase (PHD) to stabilize HIF-1α, further amplifying glycolytic signaling and establishing a positive feed-forward loop (29, 48). Upstream, mTORC1 serves as the central integrator: it boosts global translation via 4E-BP/S6K phosphorylation while simultaneously activating the SREBP2-driven mevalonate synthesis pathway and glutaminolysis, supplying epigenetic modifiers with substrates from three distinct metabolic axes (29, 49). In the tumor context, continuous metabolic input from the TME is proposed to keep the mTORC1–HIF-1α axis constitutively engaged. Sustained activation of this axis may represent the molecular node through which tumors hijack trained immunity and is hypothesized to distinguish tumor-driven from infection-driven trained immunity (50) (see Section 6.3).

Counterbalancing this positive loop is the itaconate pathway: synthesized by IRG1, itaconate induces NRF2-mediated antioxidant transcription while inhibiting SDH activity to curb fumarate accumulation (47, 51). The fumarate-to-itaconate concentration ratio may influence or contribute to the balance between inflammatory training and tolerance-like reprogramming, although the net effect appears to be stimulus-, time-, and cell-type-dependent in the published studies. In preclinical tumor models, IRG1 is frequently upregulated by the TME, skewing the equilibrium toward itaconate accumulation and suppressing antitumor training in those settings (51); tumor cells themselves may secrete itaconate via ACOD1, generating a regional immunosuppressive gradient in preclinical models. It should be noted, however, that itaconate also has context-dependent anti-inflammatory and immunometabolic effects and does not function solely as an inhibitor of trained immunity. The mevalonate-cholesterol pathway further reins in training intensity through LXR-mediated feedback, a negative-feedback mechanism that can be disrupted by aberrant accumulation of oxidized cholesterol species within the TME (49, 52).

### 6.2 Central HSPC training: a double-edged sword in the tumor context

Building on the macrophage lifespan and turnover dynamics discussed in Section 5.1, the persistence of trained immunity effects for months raises a mechanistic question: circulating monocytes have a half-life of merely 1–7 days. This kinetic discrepancy points to the bone marrow HSPC compartment as the reservoir of long-term memory (53). Peripherally activated innate immune cells release IL-1β and GM-CSF into the circulation, relaying training signals to the bone marrow microenvironment; engagement of HSPC surface receptors then triggers intrinsic transcriptional and epigenetic reprogramming programs (4, 36). This cytokine relay mechanism converts a transient peripheral stimulus into sustained myeloid output from the bone marrow: IL-1β promotes HSPC proliferation and myeloid-biased differentiation, while GM-CSF cooperatively sustains the metabolic foundation that maintains this bias (4).

On the beneficial side, HSPC training could continuously generate functionally enhanced antitumor myeloid cells, offering one speculative mechanistic explanation (among several plausible contributors) for the multi-year recurrence protection seen after intravesical BCG therapy in human bladder cancer (6). The direct causal link between HSPC reprogramming and long-term clinical protection in BCG-treated patients remains unproven; alternative explanations include sustained local inflammation, antigen-specific adaptive immunity, and repeated urothelial immune activation (3, 6, 9). On the detrimental side, sustained stimulation of the bone marrow microenvironment by tumor-derived GM-CSF, IL-6, and G-CSF may degrade central training into a sustained source of MDSCs and M2-like TAMs, as observed in animal models. C/EBPβ, the master transcription factor executing this reprogramming, establishes differential epigenetic marks at regulatory regions of self-renewal and myeloid differentiation genes, which are propagated to progeny across asymmetric divisions through histone-modification read-write mechanisms ( (37) Consequently, once chronic tumor hijacking is established, the myeloid output bias from the bone marrow may persist long after the primary tumor is removed, a concept derived largely from animal models of chronic inflammation. This enduring reprogramming is hypothesized to represent one of the fundamental features that distinguish tumor-associated trained immunity from its acute infection-driven counterpart.

### 6.3 Regulation of training polarization by TME metabolic signals

Metabolic conditions within the TME may influence myeloid-cell state and thereby modify the conditions under which trained-immunity-like phenotypes arise or persist; direct evidence for this relationship in human tumors remains limited (17). Lactic acidosis, a hallmark of the TME (54), elevates extracellular lactate to 20–40 mM, compared with 1.5–3 mM in normal tissues. After uptake into myeloid cells via MCT1 and MCT4 transporters, lactate modulates macrophage polarization through two mechanisms: it feeds the TCA cycle as a metabolic fuel, and histone lactylation participates in transcriptional regulation of macrophage polarization genes, including M2-associated programs, in non-tumor macrophage models (17, 18, 55). Whether this lactate-histone lactylation axis constitutes a durable, restimulable trained memory that persists after stimulus removal in tumor settings remains to be formally established. Hypoxia engages the HIF-1α-glycolysis feed-forward loop characterized in Section 6.1, yet the relationship follows a nonlinear dose-response in non-tumor models: moderate hypoxia (1%–5% O_2_) amplifies the training response, whereas severe hypoxia (<1% O_2_) suppresses oxidative phosphorylation and weakens the effect (13).

Extracellular ATP and its degradation product adenosine constitute a second regulatory layer. ATP amplifies pro-inflammatory signaling through the P2X7 receptor and activates the NLRP3 inflammasome. Adenosine, after engaging the A2A receptor, redirects the response toward an immunosuppressive phenotype via the cAMP-PKA-CREB axis (56). High expression of the ectonucleotidases CD39 and CD73 in the TME accelerates ATP-to-adenosine conversion, flipping the signaling polarity from pro-inflammatory to immunosuppressive. This enzymatic activity is itself a major mechanism of tumor immune escape. The energy deprivation pathway further remodels the metabolic baseline of trained immunity: AMPK inhibits mTORC1 and activates PGC-1α-driven fatty acid oxidation, shifting myeloid cells from glycolysis-dependent metabolism toward an oxidative mode (13, 57).

These TME signals form a highly coupled network. Hypoxia drives lactate production through the HIF-1α-glycolysis positive feedback described in Section 6.1. CD39 and CD73 accelerate ATP-to-adenosine conversion, reversing signaling polarity. Nutrient deprivation suppresses mTOR via AMPK while upregulating fatty acid oxidation. This signal coupling means that single-target interventions risk being offset by compensatory changes. A central unresolved challenge remains: the vast majority of mechanistic studies rely on non-tumor models, and whether the TME’s distinctive low-oxygen, acidic, lactate-rich milieu activates a response spectrum that diverges from canonical training programs remains unvalidated in human tumors. This knowledge gap directly limits the predictive power of the vaccine amplifier strategies discussed in Section 8 for solid tumor microenvironments.

### 6.4 Local training and functional plasticity of tumor-associated macrophages

The local priming or polarization of TAMs can operate independently of bone marrow HSPCs and directly shape the immune trajectory within the TME; whether this constitutes canonical trained immunity depends on demonstrating persistence after stimulus withdrawal. Single-cell sequencing in mouse tumor models has shown that β-glucan-trained TAMs maintain enhanced ROS production, pro-inflammatory cytokine secretion, and high MHC-II expression even within an immunosuppressive TME, and this phenotype remains detectable at day 14 post-tumor implantation (23). This HSPC-independent local effect serves a critical intermediate role during the early window before adaptive immunity is fully mobilized: trained TAMs produce IL-12 and IL-18, which activate NK cells and promote Th1 differentiation, providing innate-immune pre-activation that primes subsequent T cell responses (58).

The functional plasticity of TAM polarization, however, varies markedly across tumor stages. In early-stage TMEs in animal models, where M1-like signals (IFN-γ, DAMPs) dominate, trained TAMs maintain antitumor function. As tumors progress and M2-like signals (IL-4, TGFβ, M-CSF) gradually accumulate, the trained memory can be progressively rewritten toward a pro-tumorigenic direction (23, 59) (M1/M2 polarization is a simplified binary designation that does not fully capture the continuum of TAM activation states). This temporal pattern, if confirmed in human tumors, points to a potential therapeutic window for trained-immunity-based interventions; however, the optimal window remains to be established. TAMs arise from two distinct origins: tissue-resident macrophages, of embryonic origin and maintained by local self-renewal; and monocyte-recruited macrophages, continuously replenished from bone marrow HSPCs. Their relative contributions vary by tumor type and stage, and their responsiveness to training stimuli and memory durability may differ substantially. A further complication is that myeloid cells generated solely through central training can still be re-educated by the local TME upon infiltration; strategies that combine reinforcement of local TAM priming or polarization may therefore prove more resistant to this re-education. Section 7 evaluates the bidirectional antitumor and pro-tumor effects of trained immunity and establishes core constraints for vaccine safety design.

Recent single-cell analyses reveal that human macrophages encode stimulus-specific information of prior exposures, with distinct training phenotypes persisting for at least six days through differential licensing of inflammatory transcription factors and chromatin remodeling (60).

### 6.5 Integrative model and translational implications

We propose four key elements of this molecular network. mTOR-HIF-1α functions as the upstream integration hub. The fumarate/itaconate balance serves as the central metabolic-epigenetic switch. The lactate–histone lactylation pathway and the CD39/CD73–adenosine cascade constitute the TME-specific input channels. C/EBPβ-HSPC operates as the long-term memory repository. Three core features of this proposed network carry potential translational implications. The structural robustness imparted by multiple positive feedback loops and metabolic bypass routes explains why single-target interventions rarely abolish trained immunity: multi-node synergistic intervention becomes a necessity rather than an option. The dynamic equilibrium among the fumarate/itaconate ratio, the mTOR-driven anabolic arm, and the SQLE-LXR negative-feedback circuit offers tractable molecular targets for engineered modulation. The hierarchical division between central and peripheral training, meanwhile, demands that therapeutic strategies achieve precise matching in both the temporal intervention window and the spatial targeting domain. This four-layer model bridges molecular mechanism to therapeutic design: the mTOR-HIF-1α hub informs metabolic adjuvant selection; the fumarate/itaconate switch guides the choice between STING-pathway and mevalonate-pathway interventions; the lactate-adenosine input channels define TME-specific constraints that vaccine formulations must overcome; and the C/EBPβ-HSPC memory repository dictates the temporal dosing cadence. Section 8 discusses how these principles translate into actionable therapeutic designs, including BCG-based recombinant platforms and β-glucan nanoparticle delivery systems.

**Figure 2** summarizes this integrative framework. It is a conceptual synthesis: direct experimental support varies by pathway and tumor context, and the proposed connections should not be interpreted as a fully established causal network in human tumors.

**Figure 2:**
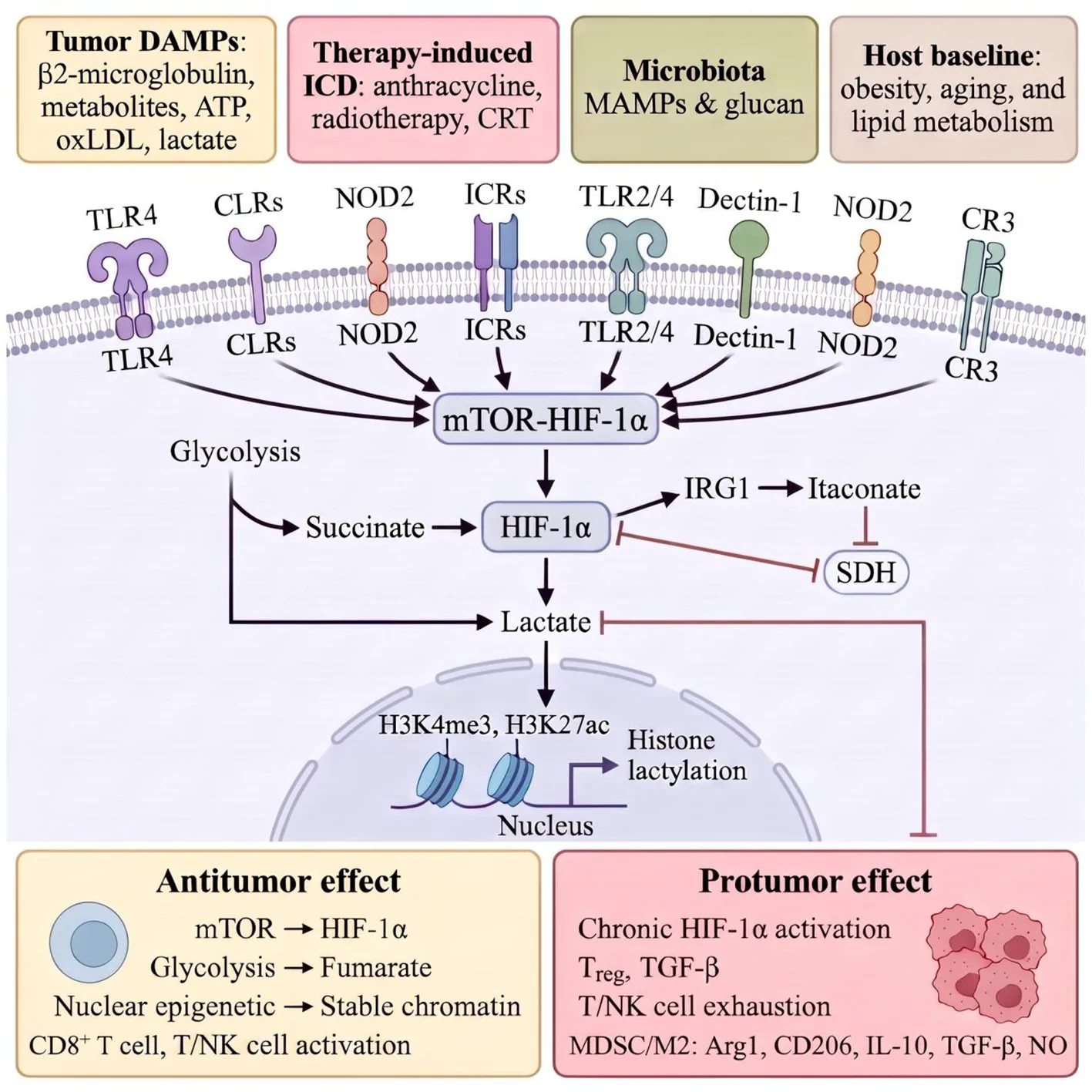
Metabolic-epigenetic coupling in trained immunity. Tumor-derived DAMPs, therapy-induced immunogenic cell death, microbiota-associated molecular patterns, and host metabolic baseline signals activate mTOR–HIF-1α signaling, driving glycolytic flux, succinate accumulation, and histone lactylation that together stabilize pro-inflammatory or immunosuppressive chromatin states.

## 7 Boundary conditions and the double-edged effects of trained immunity in the tumor microenvironment

Trained immunity cannot simply play a protective antitumor role in the TME. The TME possesses distinctive features including chronic persistent inflammation, metabolic dysregulation, and a multi-layered immunosuppressive network that can drive the same metabolic-epigenetic regulatory program toward opposing biological outcomes. A systematic dissection of this bidirectional regulation is therefore a prerequisite for developing safe and targeted trained-immunity-based antitumor interventions. Unless otherwise specified, the mechanisms discussed below are established primarily in preclinical and animal models; human clinical validation remains limited.

### 7.1 Mechanisms of trained immunity-mediated antitumor effects

The antitumor function of trained immunity is mediated by multiple parallel pathways acting in concert, with its central premise being sustained immune clearance pressure on tumor tissue driven by long-term innate immune memory (61, 62); however, these antitumor pathways lack human clinical validation.

As hypothesized from preclinical data and the single human application with established clinical benefit (intravesical BCG), durable myeloid innate memory may contribute to continuously remodeling the tumor immune microenvironment. This could provide diverse cancer vaccines with a T-cell-independent, broad-spectrum antitumor capacity. It is unlikely to be the primary source of the clinical benefit seen with BCG, however; other concurrent mechanisms include local inflammation, antigen-specific T-cell responses, and NK-cell activation. Evidence for β-glucan and other training inducers in human oncology remains preliminary.

First, direct myeloid killing capacity is substantially enhanced. BCG- or β-glucan-trained macrophages eliminate tumor cells through the NOX2-ROS and TRAIL pathways, while also strengthening antibody-dependent cellular cytotoxicity and phagocytosis (63). Kalafati and colleagues showed that β-glucan-induced innate immune training reprograms bone-marrow granulopoiesis, enhancing neutrophil ROS production and tumor infiltration and suppressing tumor growth across mouse models (63). This finding supports β-glucan-driven central training and should not be interpreted as evidence for BCG training in that experimental system. This effect likely reflects bone marrow granulopoiesis reprogramming rather than mature-neutrophil-autonomous memory, and may be amplified by BCG vaccination.

Second, trained immunity and innate priming reshape the TME cytokine network and reinforce antigen presentation. Trained macrophages secrete high levels of IL-12 and IL-18, promoting NK cell IFN-γ secretion, driving Th1 differentiation, and enhancing CD8+ T cell cytotoxic function (64). They simultaneously upregulate CXCL9 and CXCL10 to chemoattract effector T cells into tumors, while downregulating Arg1 and IL-10 to relieve immunosuppression (26). Primed or trained DCs upregulate MHC-I/II and CD80/CD86, improving antigen presentation efficiency and providing sufficient co-stimulatory signals to break T cell tolerance (65). This suggests that trained immunity could serve as a novel adjuvant, enhancing tumor vaccine immunogenicity at the innate immune level. This effect may be amplified by nano-β-glucan vaccines, a concept awaiting human validation.

Third, local priming or polarization can partially reverse the TME suppressive network. β-glucan-pretreated TAMs retain an M1-like memory phenotype even within the suppressive TME, preserving high levels of ROS and MHC-II while downregulating Arg1 and IL-10 (23). Because this effect does not depend on continuous HSPC output, peripheral *in situ* training is hypothesized to carry independent antitumor value during early tumor stages (66). By persistently promoting effector T cell infiltration, trained macrophages can partially counteract the immunosuppressive landscape of the TME (26). This effect may be amplified by LNP vaccines or local sustained-release carriers, a concept requiring human validation.

### 7.2 Pro-tumor mechanisms driven by trained immunity in the tumor microenvironment

Chronic persistent stimulation, rather than the transient pulses that define canonical trained immunity, can reroute the same metabolic and epigenetic programs toward tumor promotion (25). Two risk nodes illustrate how this shift occurs.

Sustained activation of the IL-6–STAT3 axis is the first. Trained myeloid cells under chronic stimulation maintain continuous IL-6 secretion, which drives tumor cell proliferation, EMT, and PD-L1 upregulation (27). Concurrently, MMP9 and VEGF jointly promote the vascular abnormalities characteristic of the TME (40). Pulsatile vaccine release design offers a conceptual way around this: by delivering the training stimulus in discrete bursts, it may avoid the persistent cytokine output that fuels the STAT3 feed-forward loop.

The second node operates at the hematopoietic level. When stimulation is unremitting rather than episodic, HSPCs are progressively biased toward MDSC differentiation (40), and the accompanying increase in NET release directly facilitates metastatic seeding (32). NET formation itself is not a specific marker of trained immunity, and sustained NET release should not be interpreted in isolation as maladaptive trained immunity. Pulsatile vaccine delivery may similarly curtail this outcome by preventing the chronic myelopoietic skewing that sustained stimulation otherwise induces, a concept requiring preclinical and human validation.

In both cases, tumor-promoting effects were observed to depend on prolonged continuous stimulation in preclinical models, a temporal constraint that may inform vaccine dosing design, pending human validation (67, 68).

### 7.3 Dynamic equilibrium setpoints governing the net biological effect of trained immunity

Given that both antitumor and pro-tumor pathways share overlapping molecular machinery, what determines the net biological effect? Whether trained immunity in the TME ultimately resolves as an antitumor or pro-tumor phenotype depends on a dynamic equilibrium setpoint (61), derived largely from non-tumor inflammation systems. Three regulatory dimensions are examined below, each with direct implications for vaccine engineering.

The first dimension is stimulus duration. In mouse models, acute single-dose administration may induce antitumor memory, characterized by a transient inflammatory peak followed by efficient tumor clearance; whether this translates to human tumors remains to be validated. Chronic continuous exposure, whether from sustained DAMPs release by the tumor or from factors such as long-term high-fat diet, tilts the balance toward tumor promotion through persistent IL-6–STAT3 activation and the progressive accumulation of immunosuppressive imprints (13, 17). This temporal constraint, if confirmed in humans, directly motivates hit-and-run pulsatile dosing strategies and the 7–14-day sustained-release hydrogel vaccine design: once trained memory is established, the amplifier signal is promptly withdrawn, thereby avoiding chronic pro-tumor reprogramming.

A second dimension involves cellular hierarchy. Centrally trained memory at the HSPC level is hypothesized to be durable and may resist TME-mediated reversal over short time scales, based on preclinical data, making it suited for systemic, long-acting protection. Local TAMs, by contrast, are readily re-educated by the TME, highly plastic but limited in memory stability (11, 25). A dual strategy that pairs bone marrow-targeted HDL nanoparticle vaccines (central training) with local adjuvants (*in situ* TAM training) could reconcile systemic protection with local resistance.

The third dimension is host baseline state. Obesity-associated metabolic endotoxemia constitutes a background of persistent low-grade pre-training in non-tumor human cohorts (69); aging elevates basal chronic inflammation and reduces the available training capacity (62); gut microbiota homeostasis shapes the foundational immune memory repertoire (70). These variables motivate stratified regimens. For example, pre-vaccination microbiota modulation or epigenetic priming with HDAC inhibitors to open chromatin accessibility could improve trainability in low-responder patients (67).

These three setpoints point to a principle: the bifurcation between antitumor and pro-tumor trained immunity phenotypes is substantially shaped by, but not limited to, stimulus duration, cellular hierarchy, and host baseline state. Beyond these three dimensions, additional variables modulate the response: stimulus type and receptor, tumor type and stage, local metabolic context, cellular source, microbiome composition, concurrent therapies, dose, route of administration, and host sex and genetic background. This relationship is best understood as a conceptual model that remains to be prospectively validated, rather than a fully established law. It translates into the engineering constraints that underpin the vaccine design strategies discussed in Section 8, where each of these regulatory dimensions could inform the selection and combination of vaccine components.

## 8 Trained-immunity-based anticancer interventions and vaccine platforms

### 8.1 Overview of trained-immunity-based anticancer interventions and vaccine platforms

This section focuses on translational anticancer interventions. Principles derived from prophylactic vaccines against infectious diseases are discussed only as mechanistic and design analogies: their clinical objectives, immune context, route of administration, and efficacy endpoints cannot be assumed to translate directly to therapeutic cancer vaccination. The concept of trained immunity has opened a new dimension in cancer immunotherapy that extends beyond adaptive immunity (71). Among trained immunity-based modalities, vaccine strategies and immunotherapeutic platforms (72) have become the most active area of translational research, recently reviewed comprehensively (73), as they can serve both as standalone interventions and as potentiating components of existing immunotherapies (19, 45, 74, 75). These platforms encompass: (i) interventions with established clinical use (intravesical BCG); (ii) preclinical trained immunity platforms (recombinant BCG, β-glucan nanoparticles, LNP-mRNA bifunctional vaccines); (iii) candidate trained immunity adjuvants for conventional vaccines (commensal microbiota-derived peptidases); and (iv) the authors’ proposed conceptual bifunctional vaccine design.

The core design philosophy of trained immunity vaccines departs fundamentally from the conventional antigen-adjuvant binary paradigm. Traditional vaccines need only induce antigen-specific adaptive immune memory; trained immunity vaccines must additionally activate metabolic-epigenetic reprogramming in innate immune cells, conferring broadly enhanced responsiveness against heterol **Figure 3** provides a comparative, hypothesis-oriented map of the major platform classes discussed below; it is not a clinical ranking, and the strength of evidence differs substantially across platforms. ogous pathogens or tumor cells (76). Dominguez-Andres and colleagues proposed that next-generation vaccines should comprise three components: antigen, adjuvant, and a trained-immunity amplifier that drives metabolic and epigenetic regulation (76, 77).

**Figure 3:**
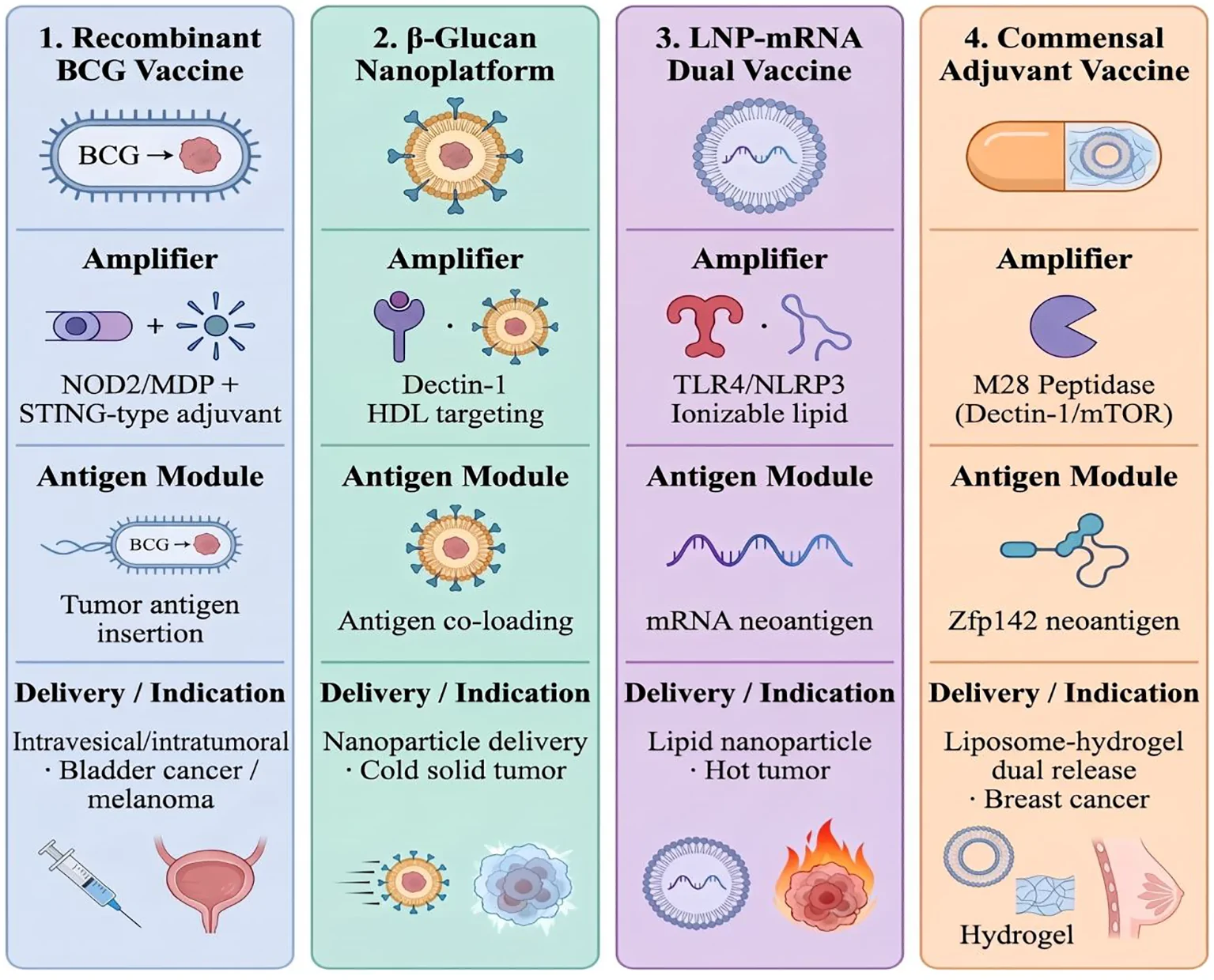
Four trained-immunity vaccine platforms. The tri-component design model comprises an amplifier, antigen module, and delivery or indication module. Illustrated platforms include recombinant BCG, β-glucan nanoparticle, LNP-mRNA dual, and commensal adjuvant vaccines.

### 8.2 Recombinant engineered BCG vaccines: systematic upgrade of the classical paradigm

#### 8.2.1 STING pathway-enhanced recombinant BCG

Intravesical instillation of wild-type BCG has been a mainstay of NMIBC treatment for over forty years. Its established efficacy reflects a multi-component local immune response that includes innate activation, antigen presentation, and adaptive T-cell-mediated immunity; trained immunity is therefore considered one contributing mechanism rather than a sole explanation for clinical benefit. Recombinant BCG approaches that incorporate innate-sensing agonists, including STING-pathway agonists, remain an actively investigated preclinical strategy. Because the specific study previously cited here could not be confirmed with consistent bibliographic metadata and a closely related report from the same research line has been retracted, no specific quantitative or mechanistic claims from that report are retained in this review. Well-controlled, independently replicated studies are required to establish whether engineered BCG can safely enhance trained-immunity-associated antitumor effects beyond those of conventional BCG.

#### 8.2.2 Antigen-chimeric BCG

Engineering BCG to express tumor antigens is a conceptually attractive way to combine microbial innate stimulation with antigen-specific adaptive immunity. BCG-associated PAMPs, including MDP, can engage innate-sensing pathways, whereas an encoded tumor antigen could in principle be processed by APCs and support tumor-specific T-cell responses. However, we did not identify a directly supporting original study for the specific claim that antigen-chimeric BCG outperforms conventional BCG and peptide vaccination in post-resection bladder-cancer recurrence models. This approach should therefore be considered a preclinical design hypothesis. Potential local applications, such as intravesical use after resection of NMIBC or intralesional delivery for accessible tumors, require disease-specific safety, antigen-expression, and efficacy testing before clinical translation.

#### 8.2.3 Attenuated non-viable bacterial derivatives

Live BCG can cause serious infection, particularly in immunocompromised patients; non-viable bacterial derivatives such as BCG cell-wall preparations or defined MDP-based formulations are therefore of interest as potentially safer innate immune stimulants. However, the references previously cited in this section do not directly support the specific claims that purified MDP alone establishes a BCG-equivalent histone-lactylation program, that low-dose intravenous delivery is established, or that intravenous CWS reduces pulmonary metastasis with superior safety in the stated melanoma and colon-cancer models. These formulations should be described as candidate preclinical tools whose trained-immunity activity, pharmacology, antitumor efficacy, and safety require direct evaluation rather than as validated alternatives to viable BCG.

#### 8.2.4 Clinical stratification and directions for improvement

The route, dose, formulation, and host context of BCG administration are likely to influence the balance between local and systemic innate immune effects. Local delivery is clinically established for NMIBC, whereas systemic or alternative routes may engage different immune compartments but cannot be assigned a fixed peripheral-versus-central training profile on the basis of current evidence. Likewise, cytokine release after ex vivo restimulation and epigenetic marks in myeloid cells are plausible exploratory measures of trained-immunity-associated responses, not validated predictors of disease-free survival in bladder cancer. Future studies should prospectively evaluate how BCG substrain, route, dose, timing, and host factors influence immune programs and clinical outcomes. For patients who do not respond to BCG, combinations with adaptive immunotherapies, including checkpoint blockade, are rational hypotheses that require controlled clinical testing; pharmacological enhancement of trainability in low responders is discussed in Section 8.7.

### 8.3 β-glucan nanoparticle delivery vaccine platforms

While recombinant BCG platforms optimize the classical mycobacterial paradigm, β-glucan offers a chemically defined alternative that eliminates infectious risk. As a natural polysaccharide component of fungal cell walls, β-glucan can induce trained-immunity-associated metabolic and epigenetic reprogramming (29). The anatomical distribution, durability, and progenitor-level effects of free versus nanoformulated β-glucan depend on formulation, dose, route, and experimental context; they should not be assumed to map rigidly onto peripheral versus central training. Nanocarrier engineering is therefore a plausible strategy to improve myeloid-cell and bone-marrow exposure, but its comparative advantage requires direct validation in each platform. In colorectal cancer models, β-glucan-induced training has been associated with macrophage epigenetic and metabolic rewiring and with enhanced recruitment or activation of effector immune cells (78).

#### 8.3.1 HDL nanobiologic (MTP10-HDL)

The MTP10-HDL nanobiologic developed by Priem and colleagues provides an important preclinical example of systemic trained-immunity induction in solid-tumor models (79). The platform uses an HDL-based nanocarrier and was reported to reshape myeloid-cell programs, suppress tumor growth, and enhance the activity of anti-PD-1 therapy in mice. These results support further investigation of systemic nanobiologic delivery as a means to counter immunosuppressive tumor microenvironments. However, the precise trafficking route, the duration and anatomical locus of training, and the optimal sequencing with immune checkpoint blockade should not be generalized beyond the experimental conditions tested; human validation is required.

### 8.4 Potential integration of trained-immunity principles into LNP-mRNA cancer vaccines

#### 8.4.1 LNP lipids as innate priming or trained-immunity-like amplifiers

The ionizable lipid component of LNP carriers has established intrinsic adjuvant activity and can trigger acute innate immune activation (80–83). In some experimental systems, LNP exposure has been associated with metabolic and epigenetic changes in myeloid cells that overlap with features of trained immunity. However, available evidence does not establish a universal TLR4/NLRP3-to-mTOR/HIF-1α-to-histone-lactylation pathway, a fixed duration of memory-like activity, or bona fide durable trained immunity in cancer patients. LNP composition can alter inflammatory and cellular responses, so its use as a trained-immunity-potentiating component should be regarded as a testable design hypothesis rather than an established mechanism. Acute innate priming and long-term trained immunity are related but distinct phenomena and should not be conflated in cancer-vaccine studies.

#### 8.4.2 A two-component mRNA vaccine design framework

Building on these properties, we propose a two-component mRNA cancer vaccine design framework as a conceptual hypothesis. The first is an antigen-encoding module: mRNA encoding tumor neoantigens or tumor-associated antigens (TAAs) is translated by host ribosomes and presented via the MHC class I pathway, activating CD8+ T cell adaptive immunity. The second is a trained immunity-potentiating module, implementable through three testable routes: (i) exploiting the intrinsic innate priming activity of the LNP ionizable lipid; (ii) co-delivering mRNA encoding trained immunity regulators such as STING agonists to reinforce innate memory inscription; and (iii) incorporating trace amounts of β-glucan or CWS components into the LNP formulation for physical co-delivery. The central advantage is that this framework could integrate innate immune memory and adaptive antitumor immunity within a single platform, but this remains to be tested in human cancer trials (79, 83–85).

#### 8.4.3 Temporal and combination strategies

The immune response kinetics of bifunctional mRNA vaccines may suggest context-dependent dosing schedules, but an optimal human schedule has not been established. The sequence from early LNP-driven innate activation to antigen expression and T-cell priming is biologically plausible, but the timing and any memory-like myeloid component require direct measurement in each formulation and disease setting. Sequential designs, such as β-glucan nanobiologic pretreatment followed by a neoantigen mRNA vaccine, are therefore conceptual strategies for preclinical testing rather than evidence-based clinical schedules. We found no published clinical evidence that melanoma mRNA vaccine trials have validated peripheral-monocyte trained-immunity markers, H3K4me3 ChIP-qPCR, or metabolic-flux measures as surrogate predictors of survival; these should be framed as candidate exploratory endpoints only.

#### 8.4.4 Clinical translation challenges

mRNA-LNP vaccines face three translational bottlenecks. Repeated dosing can induce anti-PEG antibodies that progressively reduce delivery efficiency; degradable lipids or PEG-alternative modifications offer potential workarounds. The liver preferentially sequesters LNPs, which favors systemic myeloid activation but restricts local efficacy in extrahepatic solid tumors. The dose-response relationship of ionizable lipids is non-linear: excessive doses trigger NLRP3 hyperactivation and acute toxicity, whereas doses that are too low fail to establish durable innate priming. Developing sustained-release hydrogel co-delivery systems and optimizing lipid chemistry are the key directions for overcoming these hurdles (83, 84, 86).

### 8.5 Commensal microbiota-derived trained immunity adjuvant vaccines

#### 8.5.1 Mechanism of action of the M28 peptidase adjuvant

Trained-immunity-inducing molecules can also be sourced from commensal microbiota, expanding the repertoire of candidate adjuvants. Zhou and colleagues identified an M28-family peptidase from Peribacillus frigoritolerans as an inducer of trained immunity in an infection model (87). The primary study attributes this activity to a complosome-phosphatidylcholine axis; it does not support the detailed Dectin-1/STAT3/CXCL9/CXCL10 mechanism previously stated here. In a subsequent cancer-vaccine study, M28 was incorporated into a liposome-hydrogel platform and was associated with enhanced adaptive immune responses and tumor-microenvironment remodeling in a breast-cancer model (88). The molecular mechanism linking M28 exposure to macrophage-state changes in tumors remains to be defined directly.

#### 8.5.2 Dual-phase release delivery system

In a 4T1 breast-cancer mouse model, the dual-phase liposome-hydrogel vaccine incorporating M28 and a tumor neoantigen was reported to completely inhibit tumorigenesis and pulmonary metastasis in the prophylactic setting; therapeutic administration significantly suppressed tumor progression and dissemination (88). The study also reported increased intratumoral CD4+ and CD8+ T-cell infiltration, fewer regulatory T cells, and macrophage repolarization. Because the available primary abstract does not substantiate the precise approximately 75% tumor-volume reduction previously stated here, that numerical estimate has been removed. These findings are preclinical and require independent replication and validation in therapeutic settings.

### 8.6 General design principles for trained immunity vaccines

Integrating the development experience of the four vaccine platforms discussed above, a universal three-component design model for trained immunity cancer vaccines can be distilled, mapping directly onto the core logic of vaccine development. The model comprises three modules: trained immunity amplifier, tumor antigen module, and precision delivery vehicle.

Trained immunity amplifier. The amplifier is the defining component that sets trained immunity vaccines apart from conventional vaccines: it drives the metabolic-epigenetic reprogramming of innate immune cells, writing innate immune memory. Candidate amplifiers include: microbial PAMPs such as BCG-CWS and MDP; β-glucan (Dectin-1 pathway); LNP ionizable lipids (TLR4/NLRP3 pathways), noting that LNP-mediated innate activation is primarily acute priming and its capacity for durable trained immunity induction remains unverified; and commensal microbiota-derived peptidases (Dectin-1/mTOR pathway). Amplifier selection largely determines the intensity, duration, and cellular hierarchy (peripheral versus central) of the trained immune response, and constitutes the primary design decision (89, 90).

Tumor antigen module. The antigen module initiates adaptive antitumor T-cell responses. Depending on tumor type and antigen characteristics, it can take the form of synthetic peptides, encoding mRNA, or bacterially expressed chimeric antigens. Whether the antigen and amplifier modules are co-delivered in a shared nanocarrier, administered sequentially, or delivered to spatially segregated sites determines the coupling efficiency and temporal coordination of innate-to-adaptive immune coupling.

Precision delivery vehicle. The delivery vehicle governs the spatial distribution, release kinetics, and cell-targeting specificity of both the amplifier and the antigen. Nanocarriers (HDL, liposomes, PLGA) enable bone marrow-targeted central training; hydrogels support local sustained release and *in situ* reprogramming of the cold-tumor TME; antibody conjugates permit selective M2-TAM repolarization. Vehicle choice involves a trade-off between systemic protection reach and local safety margin.

Translating this three-component model into clinical application rests on three guiding principles (1). Temporal optimization: innate immune memory establishment may require approximately 7–14 days in mouse models (human kinetics remain unknown), while adaptive effector responses may peak at 14–28 days; dosing should respect these staggered kinetics, pending human confirmation (2). Spatial stratification: central training via bone marrow progenitors is hypothesized to afford systemic, long-term protection, whereas peripheral training through TAMs and tissue-resident macrophages is expected to deliver rapid local responses; spatiotemporal coordination is conceptually preferred but unvalidated (5). Safety threshold: trained immunity occupies a narrow ledge between beneficial potentiation and detrimental hijacking. Amplifier dosing must be precisely titrated, using a hit-and-run strategy to prevent chronic stimulation from slipping into pro-tumorigenic reprogramming (91).

As a theoretical design concept without existing clinical validation, applying the three-component model to tumors of different immune phenotypes calls for differential configuration. All configurations described below are proposed hypotheses awaiting prospective clinical testing. For immune-hot tumors (e.g., MSI-H colorectal cancer, melanoma), where T-cell infiltration already exists but is functionally exhausted, the strategy centers on amplifier-driven APC reinforcement. DC-targeting mRNA-LNP platforms or β-glucan nanoformulations are conceptually preferred, delivered according to the 7-day amplifier pretreatment schedule characterized in Section 8.3.1. For immune-excluded tumors (e.g., subsets of NSCLC), in which T cells are trapped at the stromal margin, TAM-localized training via CD206-β-glucan conjugates or sustained-release hydrogel formulations would remodel the chemokine gradient (upregulating CXCL9/CXCL10). This would be combined with anti-angiogenic agents or TGF-beta inhibitors to breach the physical barrier. For immune-cold tumors (e.g., triple-negative breast cancer, pancreatic cancer), where innate-adaptive immune responses must be built from scratch, an HSPC-targeted HDL-β-glucan central training regimen combined with STING-agonistic BCG would be followed sequentially by mRNA neoantigen vaccination, pending preclinical optimization. This immune-phenotype-guided framework conceptually unifies the trigger-signal taxonomy of Section 4, the cellular hierarchy division of Section 5, and the metabolic-node targeting logic of Section 6 into a proposed clinical decision pathway (9, 92).

### 8.7 Precision vaccine strategies based on “trainability” stratification and the four-step temporal scheme

Translating trained immunity vaccines into precision clinical application calls for two operational frameworks: a trainability-based patient stratification system (93) and a temporally orchestrated sequential treatment scheme.

Individual responsiveness to training stimuli varies considerably, rooted in four biological dimensions: baseline epigenetic state, genetic polymorphisms, gut microbiota composition, and host metabolic status. As a stratification approach: patients whose baseline monocyte responsiveness falls in the upper tertile (the high-trainability group) could proceed directly to standard-dose vaccination. Where trainability is only moderate, pharmacological pretreatment with HDAC inhibitors or oral β-glucan might become necessary to elevate responsiveness (see Section 8.2.4 for the rationale). Low-trainability patients, marked by immunological fixation, pose a steeper challenge: combined epigenetic sensitizers and microbiota modulation could be deployed upfront, and v **Figure 4** is an author-proposed decision framework intended to generate testable hypotheses; it does not represent a clinically validated patient-selection or treatment algorithm. accination should be gated on reassessment confirming that responsiveness has been adequately restored, pending clinical validation.

**Figure 4:**
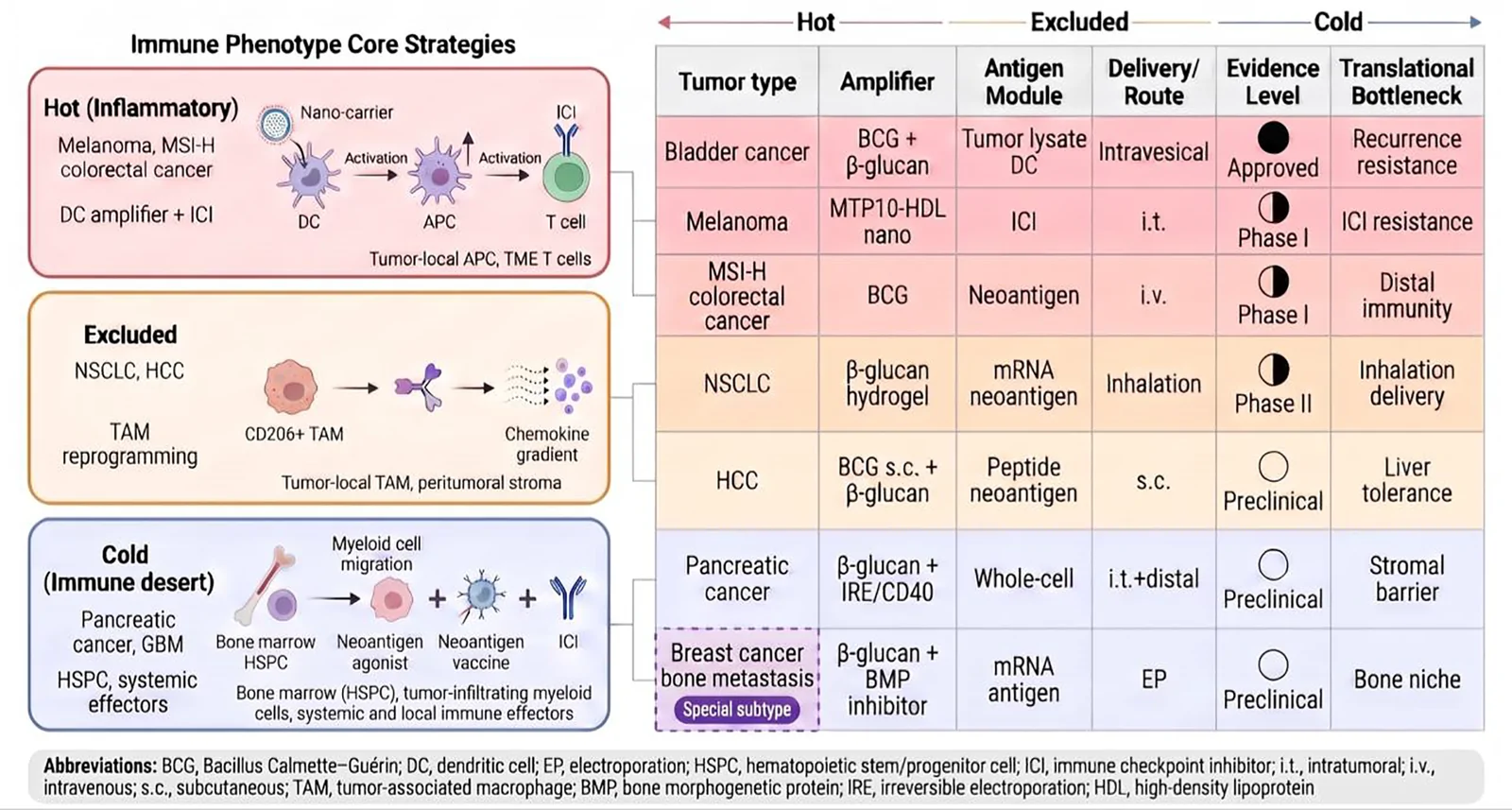
Proposed trained-immunity vaccine configuration matrix across immune phenotypes and tumor types. Immune-phenotype-driven differential strategies are presented as testable hypotheses and candidate strategies, not as clinically validated treatment algorithms. Configurations pair tumor-specific amplifier and antigen modules with the appropriate delivery route for hot, excluded, and cold tumors.

This temporally structured approach has been assessed in only one clinical setting—intravesical BCG for human bladder cancer, where trained immunity is one of several contributing mechanisms—and in one preclinical setting, mouse models combining MTP10-HDL with anti-PD-1 therapy. All other schedules remain experimental.

### 8.8 Evidence hierarchy framework and platform evidence levels

To help readers distinguish the strength of evidence underlying each claim, we apply the following five-level hierarchy throughout this review. Clinical evidence denotes interventions with demonstrated efficacy in human cancer patients. Human immunological evidence refers to observations made in humans (including healthy volunteers) that support a biological mechanism but have not been linked to a tumor outcome. Preclinical tumor evidence derives from animal tumor models. Evidence extrapolated from infectious/inflammatory models comes from non-tumor human or animal studies and is assumed, not proven, to apply to cancer. Authors’ proposed framework indicates a conceptual or therapeutic design put forward by us for future testing. **Table 2** summarizes the evidence level for each trained-immunity-based platform discussed.

**Table 2:** Evidence levels of trained-immunity-based cancer vaccine platforms.

Platform/intervention	Evidence level	Key supporting observations	Current limitations/needs
Intravesical BCG for NMIBC	Level 1: Clinical evidence	Reduced recurrence and progression in human bladder cancer; established standard of care	Limited to NMIBC; mechanistic link to trained immunity inferred
BCG-induced trained immunity in circulating monocytes/HSPCs	Level 2: Human immunological evidence	Sustained cytokine responses and H3K4me3 marks after BCG vaccination in healthy volunteers	Observations mostly in healthy volunteers; correlation with tumor outcome unvalidated
Engineered recombinant BCG (STING-BCG, antigen-chimeric BCG)	Level 3: Preclinical tumor evidence	Enhanced antitumor effects in mouse models; proof-of-concept only	No human tumor data
β-glucan nanoparticles (e.g., MTP10-HDL)	Level 3: Preclinical tumor evidence	Central HSPC training and synergy with anti-PD-1 in mouse melanoma; 7–14 day kinetics observed	No human cancer trial data
LNP-mRNA as trained-immunity amplifier	Level 4 (extrapolated) + Level 5 (proposed framework)	LNP lipids induce trained immunity in non-cancer settings; bifunctional cancer vaccine design is conceptual	Cancer-specific data lacking; human dosing kinetics unvalidated
Commensal microbiota-derived peptidase adjuvants (M28)	Level 3: Preclinical tumor evidence	Mouse model data showing enhanced trained immunity and antitumor effects	Limited platform; human data absent
Immune-phenotype-guided differential vaccine configuration	Level 5: Authors’ proposed framework	Conceptual matching of hot/excluded/cold tumor phenotypes to platform types; based on preclinical inference	No clinical validation; requires prospective trials

BCG, Bacillus Calmette-Guérin; HDL, high-density lipoprotein; HSPC, hematopoietic stem and progenitor cell; LNP, lipid nanoparticle; NMIBC, non-muscle-invasive bladder cancer.

## 9 Clinical translational evidence for trained immunity-based anticancer therapy

To date, no large-scale, randomized, prospective clinical trial has been designed with trained immunity as the primary mechanistic endpoint or therapeutic target. The evidence base therefore consists of a patchwork of established clinical practices (most notably BCG), repurposed immunomodulators, observational biomarker studies, and preclinical platforms. **Table 3** provides a systematic overview of this landscape, distinguishing interventions by evidence level and trial category.

**Table 3:** Evidence landscape of trained-immunity-based cancer interventions.

Intervention	Target/receptor	Main innate cells	Central/peripheral training	Cancer type	Evidence level	Key outcome	Main limitation
BCG (intravesical)	TLR2/4, NOD2, multiple PRRs	BCG (Level 1); BCG + β-glucan combination (Level 3, preclinical)	Level 1 (BCG standard of care, >40-year clinical data); Level 3 (combination)	NMIBC	Human clinical (standard of care)	Reduced recurrence/progression	Mechanism attribution contested; strain/dose variability
Recombinant STING-BCG	STING pathway, c-di-AMP	WGP/MTP10-HDL + ICI (Level 3, preclinical)	Level 3 (preclinical proof-of-concept); clinical Phase II data pending direct supporting references	Bladder cancer (preclinical)	Preclinical animal models	Enhanced trained immunity, improved tumor control	No human clinical data
β-glucan (systemic/nanoparticle)	Dectin-1, CR3	Monocytes, macrophages	Level 3 (preclinical proof-of-concept)	Various solid tumors (preclinical)	Preclinical (*in vivo*)	Reduced tumor growth, potentiated ICB	No human cancer trials; formulation variability
MTP10-HDL nanobiologic	β-glucan/Dectin-1 on bone marrow progenitors	Monocytes, macrophages	Level 3 (preclinical proof-of-concept)	Melanoma (preclinical)	Preclinical (*in vivo*)	Tumor suppression, checkpoint inhibition synergy	No clinical data to date
MDP/CWS derivatives	NOD2	Monocytes, macrophages	Level 3 (preclinical)	Bladder cancer (historical)	Limited human data; mostly preclinical	Mixed immunomodulatory effects	Toxicity; limited durable trained immunity evidence
LNP-mRNA cancer vaccines	TLR7/8, NLRP3 (innate sensors)	Monocytes, macrophages, DCs	Level 3 (preclinical)	Melanoma, NSCLC, pancreatic, etc.	Clinical Phase I-III (T-cell responses)	T-cell priming, durable adaptive immunity	Bona fide durable trained immunity unproven in cancer patients
Microbiota-derived M28 peptidase	Complosome-phosphatidylcholine axis	Monocytes, macrophages	Peripheral	Breast cancer (4T1 preclinical)	Preclinical (*in vivo*)	Complete suppression of primary tumor and metastasis	Human studies pending
Cytokine-trained monocytes (IFN-γ, GM-CSF, IL-1β)	Cytokine receptors	Monocytes, macrophages	Peripheral	Various (preclinical)	*In vitro* and animal models	Enhanced antigen presentation, T-cell priming	Heterogeneity; no validated biomarkers

BCG, Bacillus Calmette-Guérin; BMP, bone morphogenetic protein; CWS, cell-wall skeleton; DC, dendritic cell; HSPC, hematopoietic stem and progenitor cell; ICB, immune checkpoint blockade; IL, interleukin; IRE, irreversible electroporation; LNP, lipid nanoparticle; MDP, muramyl dipeptide; NMIBC, non-muscle-invasive bladder cancer; NSCLC, non-small cell lung cancer; PoC, proof of concept; TLR, Toll-like receptor; WGP, whole glucan particles.

These categories are mapped onto the five-level evidence hierarchy of Section 8.8 to avoid conflating established clinical practice with investigational platforms. Clinical evidence (Level 1) covers established clinical use with retrospective trained-immunity associations (principally BCG); Human immunological evidence (Level 2) comprises prospective studies in which trained immunity is the explicit primary endpoint; Preclinical tumor evidence (Level 3) covers conventional oncology trials that incidentally included immune-monitoring endpoints; Evidence extrapolated from infectious/inflammatory models (Level 4) encompasses studies in non-tumor settings; and Authors’ proposed framework (Level 5) encompasses conceptual designs. The near-absence of Level 2 trials represents a translational gap—most current evidence remains observational (Level 1), incidental (Level 3), or preclinical (Level 4).

### 9.1 Differential responses across solid tumor types and their immune microenvironmental basis

Preclinical and limited clinical experience with trained immunity-based interventions reveals a tumor-type-specific response landscape. Note that the most comprehensive clinical evidence to date comes from hematological malignancies (94); extrapolation to solid tumors remains largely hypothetical. In pancreatic ductal adenocarcinoma, a Phase II trial combining irreversible electroporation with oral beta-glucan demonstrated improved disease-free interval (median 18 months) and overall survival (median 32.5 months), with trained immunity signatures confirmed by mass cytometry at 12 months post-procedure (95). Each malignancy presents a distinct combination of a candidate strategy and its attendant bottleneck, shaped by its unique immune microenvironment.

Bladder cancer, the only tumor type with established human evidence that trained immunity contributes to clinical efficacy—in concert with local inflammatory responses and specific T-cell-mediated immunity, and memory B cells within mature tertiary lymphoid structures have emerged as independent predictors of recurrence-free survival (96)—is suited to intravesical BCG instillation; single-cell RNA sequencing of BCG-treated bladders has identified divergent macrophage-regulated T cell states that distinguish responders from non-responders, with Th17-like Th1 cytokines serving as predictive biomarkers (97); combining it with β-glucan remains a preclinical concept.

These divergent profiles reflect fundamental differences in tumor metabolic context, myeloid infiltration density, and baseline innate immune competence—variables that collectively determine whether a given trained-immunity platform can achieve therapeutic traction in a specific tumor type.

The response patterns across these tumor types suggest a conceptual three-tier design logic—grounded in immune phenotype and myeloid infiltration—that we propose as a theoretical framework for hypothesis generation; this framework has not been clinically validated and should not be interpreted as an established treatment algorithm.

## 10 Discussion

Trained immunity, the capacity of innate immune cells to acquire long-lasting functional reprogramming after an initial stimulus, offers a distinct operational dimension for tumor immunotherapy, one that provides a complementary axis to adaptive immunity. The four vaccine platforms examined in this review occupy complementary positions along this dimension, each with characteristic strengths and unresolved bottlenecks. Recombinant BCG carries the strongest clinical evidence base, grounded in decades of use in NMIBC. Its trained-immunity effects, however, arose empirically rather than by rational design. Strain heterogeneity, constrained delivery routes, and systemic toxicity cap further optimization. β-Glucan nanoparticle delivery takes a different route to the same goal. HDL carriers enable bone marrow–targeted administration, and animal models show reprogramming of systemic immunity from the HSPC level. However, human pharmacokinetic and safety data are not yet available. LNP-mRNA platforms add further flexibility by combining antigen-encoding capacity with tunable adjuvanticity, potentially driving innate trained immunity and adaptive antitumor responses in parallel. However, the innate immune activation patterns of mRNA-LNP in cancer patients remain poorly characterized, and the durability of trained-immunity induction has yet to be verified. Microbiota-derived adjuvants round out the portfolio with favorable safety profiles and systemic immune modulation via the gut–immune axis. Yet disentangling the multicomponent, multipathway network effects and establishing a causal link between individual microbial products and trained immunity pose an ongoing challenge. Across all four platforms, a translational bottleneck persists: achieving sufficient targeting and controllability without sacrificing the broad, non-specific protection that distinguishes trained immunity from antigen-specific strategies.

The bidirectional effects of trained immunity in tumors are not a fixed biological property; they can be precisely modulated through dosing schedules and delivery strategies. The dynamic balance between pro-inflammatory and tolerogenic directions is governed by three factors: the metabolic milieu of the tumor microenvironment (TME), dosing timing relative to other interventions, and the hierarchical level of target cells. Lactate accumulation, hypoxia, and lipid overload tend to push trained immunity toward tumor-promoting polarization, whereas pretreatment with trained-immunity inducers before immune checkpoint blockade may outperform concurrent dosing by allowing innate immune baselines to reset before adaptive responses are unleashed. At the cellular level, central training at the HSPC stage produces systemic, durable reprogramming with a slower onset, while local training of tissue-resident macrophages acts more rapidly but remains more susceptible to immediate metabolic cues from the surrounding microenvironment. These observations carry direct implications for clinical trial design: future trained-immunity interventions should incorporate “directional control” as a pre-specified endpoint rather than merely monitoring safety. Pre-treatment baseline immune phenotyping, temporal optimization of dosing windows, and longitudinal post-treatment monitoring of trained-immunity biomarkers together constitute a clinical framework for transitioning from empirical administration to controllable immune training.

The most significant gap in the current evidence base is the near-absence of large-scale, prospective, randomized clinical trials designed with trained immunity as a primary therapeutic target or mechanistic endpoint. The established clinical benefit of BCG in NMIBC (Level 1 evidence) was identified through empirical use, and subsequent mechanistic studies linking this benefit to trained immunity are largely observational or derived from immunological data in healthy volunteers. Trials that explicitly target trained-immunity pathways (Level 2) remain scarce, small, and exploratory. In most contemporary oncology trials (Level 3), innate immune phenotyping is collected as an incidental measurement rather than a predefined primary endpoint, precluding causal inference about the contribution of trained immunity to clinical outcomes. The majority of emerging platforms (Level 4) are still confined to preclinical models. This review therefore constitutes a translational roadmap rather than a portfolio of validated clinical regimens. The gap between mechanistic understanding and prospective clinical evidence remains the most significant obstacle facing the field (98–100).

On the basis of the preceding analysis, we propose three prioritized research directions. The first concerns immune-phenotype-driven stratified deployment. Immune-desert (cold) tumors may benefit from central training at the HSPC level combined with mRNA neoantigen strategies to reprogram systemic immune baselines from the bone marrow outward. Immune-excluded tumors call for local priming at the tumor-associated macrophage (TAM) level combined with chemokine remodeling to direct trained-immunity effects into the TME. Immune-inflamed (hot) tumors are candidates for dendritic cell–targeted enhancement in synergy with immune checkpoint inhibitors to maximize existing adaptive responses. This stratification framework is a theoretical design concept only and must not be interpreted as reflecting existing clinical evidence. No prospective trial has validated these proposed immune-phenotype-driven configurations, and their clinical feasibility remains entirely untested. A second direction involves rethinking clinical trial design. Because trained-immunity vaccines confer heterologous protection, efficacy may depend on the degree of immune developmental reprogramming rather than antigen-specific responses. If confirmed in human cancer trials, a conceptual regulatory shift would be necessary. Efficacy assessment may need to move from antigen-specific endpoints toward innate immune memory surrogates. These surrogates—H3K4me3 ChIP-qPCR, metabolic flux analysis, cytokine recall responses—would require analytical and clinical validation before regulatory acceptance. The third direction addresses spatiotemporal coordination of central and peripheral training. Nanoparticle delivery vehicles can link bone marrow targeting with local sustained release, enabling sequential regimens of “central reprogramming first, peripheral consolidation second.” Technical feasibility has been demonstrated in animal models. However, the pharmacokinetic–immunodynamic matching required for human application remains to be established.

Several limitations should be noted. First, as a narrative review, this work is subject to selection bias: the literature search was not systematic, study inclusion relied on author judgment, and relevant studies may have been inadvertently omitted despite our structured search strategy. Second, a substantial proportion of mechanistic evidence in the trained-immunity field derives from *in vitro* and animal models, and extrapolation to the human tumor context warrants caution. Third, the role of innate lymphoid cells (ILC1/2/3) in tumor-associated trained immunity remains unclear. Although ILC subsets are recognized as important regulators of tumor immunity, systematic studies linking ILCs to trained immunity are still in their infancy. Potential mechanisms—HSPC reprogramming, local priming, or cytokine-mediated functional adaptation—remain to be investigated. This review therefore focuses on cell lineages with more established mechanistic evidence; ILCs represent an important but currently underexplored frontier. Fourth, trained-immunity biomarkers are technically feasible but have not reached analytical or clinical validation standards in oncology. Several factors impede the translation of trained immunity biomarkers into bedside surrogate markers: heterogeneity in sample sources; lack of standardized restimulation conditions; absence of consensus reference stimulants or time windows; the inability of bulk H3K4me3 measurements to distinguish epigenetic reprogramming from cell-composition shifts; and confounding of baseline immune status by comorbidities, infections, obesity, and medication. No validated clinical decision thresholds have been established, nor has a standardized composite trained-immunity index been formulated. Single-cell multi-omics and machine-learning-based stratification offer potential avenues to overcome these barriers, but their clinical utility awaits prospective validation.

## 11 Conclusion

This review traces trained immunity’s regulatory network in tumor immunology through four lenses: triggers, metabolic-epigenetic reprogramming, cellular diversity, and therapeutic targeting, alongside a systematic analysis of four vaccine platforms built on this principle. Unlike conventional transient innate activation, bona fide trained immunity has the potential to provide longer-lasting functional reprogramming, although durability varies by stimulus, cell type and host context. TLR and STING agonists can also induce durable immune remodeling or adaptive immune memory in certain settings, and trained-immunity-inducing stimuli may conversely produce short-term, heterogeneous, or tolerance-like outcomes. From this analysis, three areas for advancing these vaccines come into focus. Immune-phenotype-driven differential configuration is one: HSPC-level central conditioning plus mRNA neoantigens for cold tumors, TAM-level local priming plus chemokine remodeling for excluded tumors, and DC-targeted amplifiers plus ICI synergy for hot tumors—all of which are theoretical (**Table 4**) design concepts requiring prospective clinical validation before any clinical application can be considered. Another is adaptive clinical trial designs that could, in principle, adopt innate immune memory surrogates—including H3K4me3 ChIP-qPCR, metabolic flux, and cytokine recall responses—as alternative endpoints, provided these biomarkers achieve analytical and clinical validation. A third concerns spatiotemporal coordination of central and peripheral conditioning, where nanoparticle carriers could bridge bone marrow targeting with local sustained release. Progress along these directions could move innate immunity beyond a supportive role, offering a perspective distinct from adaptive immune strategies for ICI resistance and cold-tumor intractability, provided human trials confirm the preclinical and conceptual evidence presented here.

**Table 4:** Proposed differential response profiles and evidence levels of trained-immunity vaccines across solid tumor types. The listed regimens are candidate strategies, not established treatment recommendations.

Tumor type	Immune phenotype	Proposed candidate strategy	Evidence level	Translational bottleneck
Bladder cancer	Moderately infiltrated	BCG (Level 1); BCG + β-glucan combination (Level 3, preclinical)	Level 1 (BCG standard of care, >40-year clinical data); Level 3 (combination)	~50% non-response; predictive biomarkers lacking
Melanoma	Inflamed (hot)	WGP/MTP10-HDL + ICI (Level 3, preclinical)	Level 3 (preclinical proof-of-concept); clinical Phase II data pending direct supporting references	Reinforces existing responses rather than *de novo* priming
Lung cancer	Intermediate/excluded	Inhaled β-glucan	Level 3 (preclinical proof-of-concept)	Mucosal route restricts coverage of extrapulmonary sites
Pancreatic cancer	Desert (cold)	IRE (irreversible electroporation) + CD40 + PI3-β-glucan	Level 3 (preclinical proof-of-concept)	*De novo* infiltration with risk of pro-tumorigenic hijacking
Hepatocellular carcinoma	Immune-tolerant	BCG/β-glucan	Level 3 (preclinical)	Tumor-type-specific metabolic regulatory constraints
Breast cancer (bone metastasis)	Bone niche	β-glucan + BMP (bone morphogenetic protein) inhibition	Level 3 (preclinical)	BMP-mediated suppression of HSPC reprogramming

Finally, several caveats must be kept in mind. BCG remains the platform with the strongest clinical evidence, though trained immunity is one of several contributing mechanisms to its clinical efficacy; most other trained-immunity-based platforms are still predominantly preclinical. Trained immunity is not intrinsically beneficial; its net effect depends on context and may include inflammatory or tolerogenic consequences. Clinical translation will therefore require reproducible biomarkers and rigorous safety monitoring. Specific sequential regimens, including optimal combinations, dosing, and scheduling, remain to be tested in prospective clinical trials.
